# A population-based analysis of hematological malignancies from a French-West-Indies cancer registry’s data (2009–2018)

**DOI:** 10.1186/s12885-023-11666-9

**Published:** 2023-12-06

**Authors:** Rémi Houpert, Thierry Almont, Rostom Belahreche, Mamadi Faro, Jennie Okouango, Mylène Vestris, Jonathan Macni, Olivier Pierre-Louis, Christelle Montabord, Murielle Beaubrun-Renard, Naby Soumah, Martial Boisseau, Jacqueline Véronique-Baudin, Clarisse Joachim

**Affiliations:** 1 Oncology Hematology Urology Department, Oncology Research & Development Unit (UF3596), University Hospital of Martinique, Fort-de-France, Martinique; 2 Hematology Unit, Oncology Hematology Urology Department, University Hospital of Martinique, Fort-de-France, Martinique; 3 Oncology Hematology Urology Department, General Cancer Registry of Martinique (UF1441), University Hospital of Martinique, Fort-de-France, Martinique; 4 Sciences Technologies Environment Department, Cellular Biology Physiology and Pathology, West Indies University, Pole of Martinique, Martinique; 5 Oncology Department, University Hospital of Martinique, Fort-de-France, Martinique

**Keywords:** Lymphoma, Non-Hodgkin Lymphoma, Multiple Myeloma, Epidemiology, Hematological Malignancies, Caribbean

## Abstract

**Background:**

A worldwide increased incidence of HM has been marked in recent decades. Therefore, to update epidemiological characteristics of HM in a French West Indies territory, we have performed analysis through Martinique's population-based cancer registry database.

**Methods:**

We included cancer case data, from 2009–2018, coded in strict compliance with international standards set by International Agency for Research on Cancer. We calculated standardized incidence rates, cumulative rate (ages 0–74), and temporal trends for cases and deaths using the global population standard, by sex and five age group. Mortality rates were obtained from the French Epidemiology Center on Medical Causes of Death (CépiDc).

**Results:**

One thousand forty seven new cases and 674 deaths from HM were recorded, of which 501 MM (47.8%), 377 LMNH (36%), 123 LAM (11.8%), and 46 LH (4.4%) were reported in both sexes. MM is one of the hematological malignancies with the highest incidence in Martinique among men. Temporal trends of incidence rates for all HM decreased overall in both sexes, except for MM in men. There is significant variability in mortality rates for both sexes. In addition, over the period, the temporal trends of mortality rates for all HMs has decreased overall. Gender-specific rates, between 2009 and 2018, showed that all lymphoid HM have a multimodal distribution curve that increased with age.

**Conclusions:**

Characteristics of HM in Martinique over the reporting periods differ from mainland France. Higher incidences have been observed, particularly for MM, and non-significant sub-mortality is observed compared to mainland France. Moreover, temporal distribution of mortality and incidence trends had decreased over the reporting periods except for MM. Our results showed similarities with African-Americans groups in United States and in particular an equivalence in the frequency distribution of diagnosed HM. However, SMR remains lower compared to US black ethnic groups. Our results contributed to expanding knowledge on the epidemiology of HM with Caribbean data.

## Introduction

Hematological malignancies (HM) are neoplasia developed at the expense of hematopoietic tissue and lymph nodes. According to WHO International Classification of Diseases for Oncology 2001, this includes all syndromes that develop from bone marrow stem cells. They may present clinically as leukemias, solid tumors called sarcomas or lymphomas [1, 2].

There would be an East–West gradient in geographical distribution and disparities, especially in developed countries. Asian countries such as Korea [3], Singapore, and China had lower incidence and mortality rates than Western countries [4]. Within a country, disparities could also be observed among ethnic groups, such as in metropolitan France or the United States, with lower incidences in particular among Asians or Caucasians and higher incidences among African-descent [5–9].

Recent decades’ data in the literature have shown an overall increase in incidence and mortality rates for HM, with age in both sexes, but with notable geographical distributions and disparities. According to the GLOBOCAN 2018 statistics [10], these worldwide rates would be significantly higher than the GLOBOCAN 2012 statistics [11]. Each category had heterogeneous evolutionary patterns, prognosis, patterns of occurrence, and global distribution frequencies.

Martinique, French Caribbean Island whose population is mainly of African origin, had no exception to the rule. General Cancer Registry of Martinique (GCRM) had already recorded an increase in standardized incidence ratios during the period 2007–2014 [9].

In order to update epidemiological data and characteristics of HM, we have performed, through the GCRM database, an analysis of HM between 2009 to 2018.

## Materials and methods

### Population and design

This retrospective study included all cases registered by the GCRM of diagnosed HM (ICD-O-3/ codes:9590–9597, 9670–9719, 9724–9729, 9832–9838, 9650–9667, 9731–9734, 9760–9764, 9840, 9860, 9861, 9866, 9867, 9870–9874, 9891–9931, 9984, 9805, 9806–9809, 9865, 9869, 9911, 9898) between January 1, 2009, and December 31, 2018.

### Data sources/collection

Data were recorded in the GCRM in strict compliance with international standards set by International Agency for Research on Cancer, French FRANCIM network, and European Network of Cancer Registries (ENCR). Registry procedures and data quality control, through data cross-checking and analysis across all available data sources, are in full accordance with national and international guidelines for cancer registries and are approved by the General Data Protection Regulation (GDPR). GCRM provides high-quality information on cancer in Martinique and collaborates with various local organizations to provide a complete data collection circuit.

Incidence data for HM were classified into four categories and presented by sex: (i) Multiple Myeloma and Immunoproliferative diseases (MM), (ii) Non-Hodgkin's malignant lymphoma (LMNH), (iii) Acute myeloid leukemia (LAM), and (iv) Hodgkin's lymphoma. This classification within the FRANCIM network is a particularity of the French Cancer Registries in the Caribbean area. It was adopted because of the over-incidence of MM compared with France.

Data on patients' deaths in Martinique were obtained from the French Epidemiology Center on Medical Causes of Death (CépiDc), ensuring complete information on deaths. Mortality data are coded according to the 10th International Classification of Diseases, CIM-10 (Hodgkin's disease and lymphomas: C81-C86, Other malignant tumors of lymphoid and hematopoietic tissues: C88, C90, C96, and Leukemia: C91-C95).

### Statistical analysis

Patient characteristics are described as mean ± standard deviation for quantitative variables. Descriptive analysis was performed for all the HM over the period 2009–2018. Age-specific incidence rates of HM were assessed in the following five age groups: “0–4”, “5–9” “10–14”, “15–19”, “20–24”, “25–29”, “30–34”, “35–39” “40–44”, “45–49”, “50–54”, “55–59”, “60–64”, “65–69”, “70–74”, “75–79”, “80–84”, “85 and over”. Incidence standardized rates were calculated as the observed number of the main HM cases divided by the number of population over the period 2009–2018 (with 95% confidence intervals). Mortality rate were calculated as the ratio of the number of deaths during the year to the average total population for the year. We have calculated the cumulative rate, which is the sum, for each year of age, of the age-specific incidence rates from birth to age 74 for the 0–74 rate. It can be interpreted as an approximation of the cumulative risk. Cumulative risk is the risk that an individual would have of developing a hematological malignancy during the 0–74 period if no other cause of death were involved [12]. Standardized rates were calculated using the standard world population of WHO as standard. Incidence and mortality rates were expressed in person-time.

In addition, temporal trends of incidence and mortality rates were performed by sex across the study period for the main HM.

All analyses were performed using STATA version 18 (Stata Corp., College Station, Texas, USA).

## Results

**Fig. 1:**
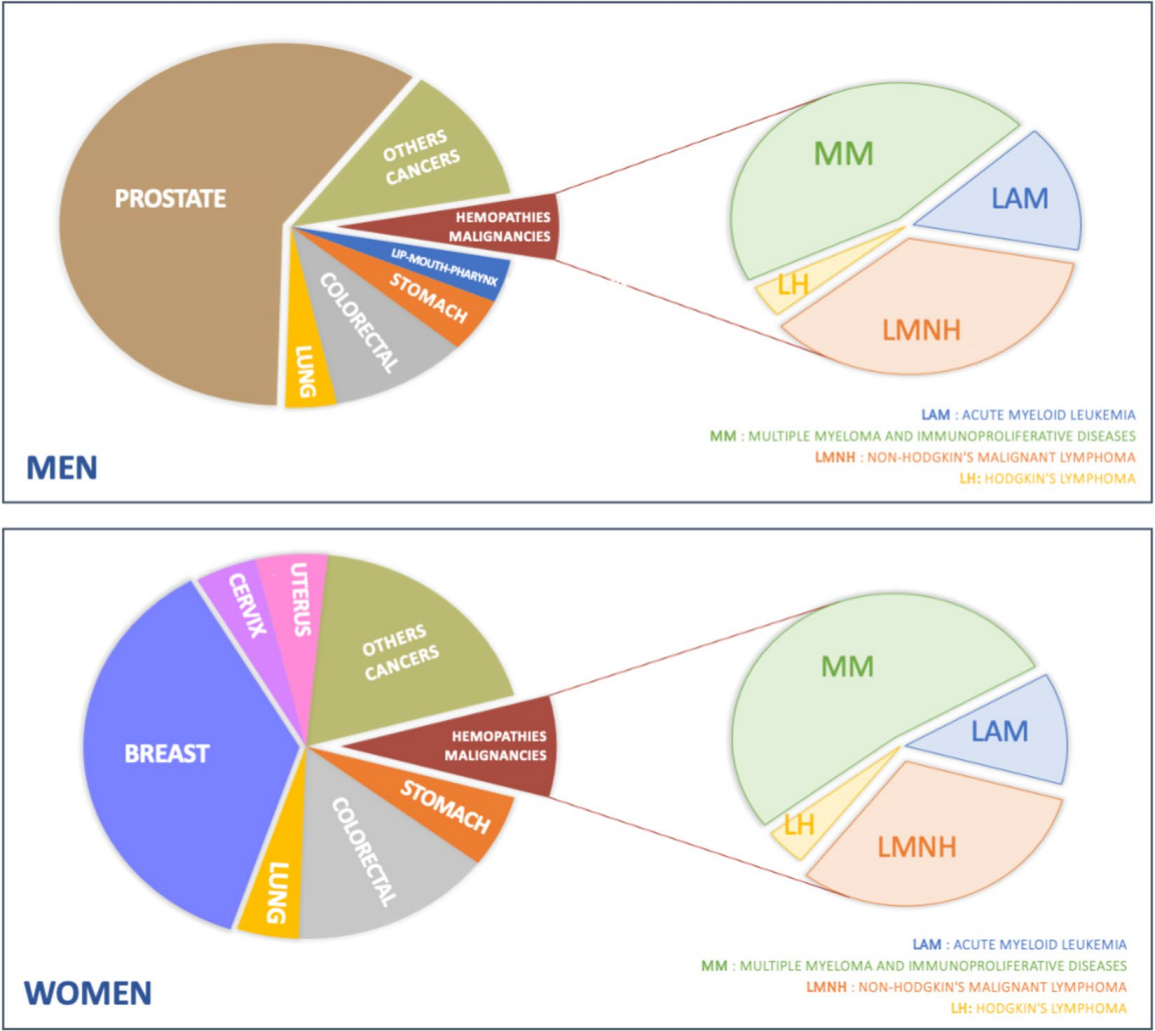
Distribution of Hematological Malignancies according to cancer cases in Martinique, by sex (2009–2018)

In Martinique, over the period 2009–2018, 1047 cases of HM (50.3% in men with 527 new cases and 49.6% in women with 520 new cases) were diagnosed (Table 1, Fig. 1), and 674 deaths were recorded (53.4% in men with 360 deaths and 46.6% in women with 314 deaths) (Table 2).

**Table 1:** All Hematological Malignancies in Martinique by sex (2009–2018): median age at diagnosis, annual number of new cases, cumulative rate during the age period 0–74 and world standardized incidence rate (TSM) with 95% confidence intervals (CI) (*n* = 1047)

	Median Age (range)	**Incidence**			Total (n)
		New cases (n)	TSM^a^ [CI]	Cumulative rate^b^ [CI]	
**HEMATOLOGICAL MALIGNANCIES**					**1047**
**Multiple myeloma and immunoproliferative diseases**					501
Men	70 [34–97]	251	6.68 [5.78; 7.56]	0.77 [0.76; 0.77]	
Women	73 [27–97]	250	4.99 [4.29; 5.69]	0.60 [0.59; 0.60]	
**Non-Hodgkin's malignant lymphoma**					377
Men	68 [4–77]	186	5.93 [4.98; 6.88]	0.66 [0.65; 0.66]	
Women	61 [61]	191	5.05 [4.25; 5.85]	0.56 [0.55; 0.56]	
**Acute myeloid leukemia**					123
Men	72 [11–95]	69	2.19 [1.6; 2.79]	0.21 [0.20; 0.21]	
Women	72 [1–98]	54	1.38 [0.94; 1.83]	0.16 [0.15; 0.16]	
**Hodgkin's lymphoma**					46
Men	39 [9–87]	21	1.23 [0.65; 1.8]	0.09 [0.09; 0.09]	
Women	33 [13–75]	25	1.36 [0.79; 1.94]	0.10 [0.09; 0.10]	

^a^World standardized rate: rates are standardized on the age structure of the world population. They are expressed per 100.000 person-years

^b^The cumulative rate is the sum over each year of age of the age-specific incidence rates, taken from birth to age 74 for the 0–74 rate

**Table 2:** All Hematological Malignancies in Martinique by sex (2009–2018): annual number of new deaths, world standardized mortality rate (TSM) (*n* = 674)

	**Mortality**		Total (n)
	Deaths (n)	TSM^a^	
**HEMATOLOGICAL MALIGNANCIES**			**674**
**Hodgkin's disease and lymphoma (C81-C86)**			165
Men	95	6.02	
Women	70	3.23	
**Leukemia (C91-C95)**			249
Men	134	8.43	
Women	115	5.39	
**Other Malignant Tumors of Lymphoid and Hematopoietic Tissue (C88, C90, C96)**			260
Men	131	8.64	
Women	129	6.07	

^a^World standardized rate: rates are standardized on the age structure of the world population. They are expressed per 100,000 person-years

SOURCE: https://opendata-cepidc.inserm.fr/

### Incidence of Hematological Malignancies in Martinique

**Fig. 2:**
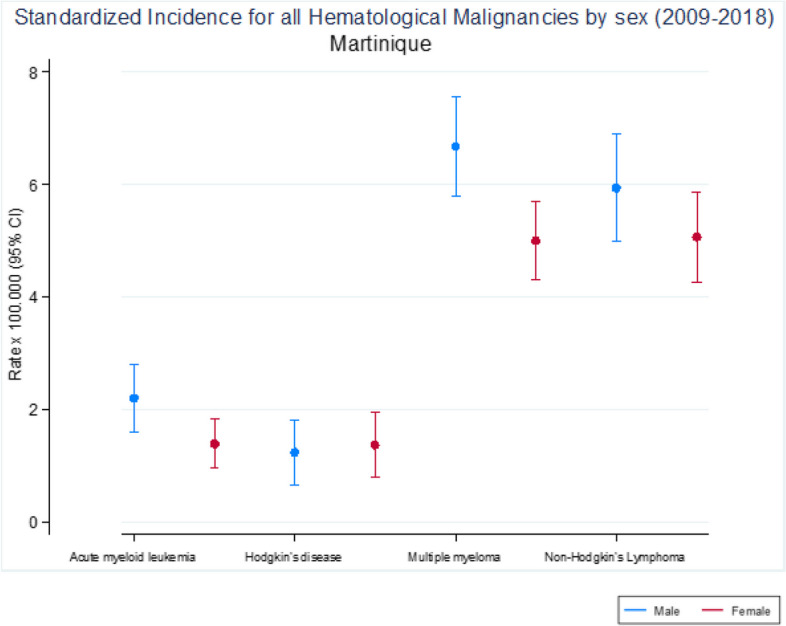
Standardized incidence for all Hematological Malignacies by sex, in Martinique (2009–2018)

Five hundred one MM (47.8%), 377 LMNH (36%), 123 LAM (11.8%), and 46 LH (4.4%) were recorded in both sexes (Table 1, Fig. 1). Incidence of hematological malignancies is higher in men, with rates close to 6 per 100,000 for MM and LMNH, compared to 5 per 100,000 for women. LAM and LH are less frequent, with meager standardized incidence rates below 2 per 100,000 (Fig. 2).

**Fig. 3:**
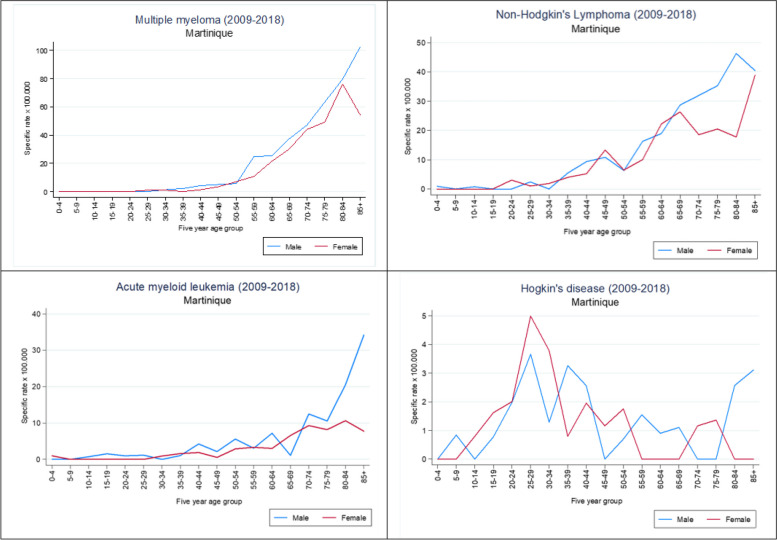
Age and Sex-specific Structure rate for all Hematological Malignancies in Martinique (2009–2018)

MM are one of the HM with the highest incidence (6.68 per 100,000 person-years in men and 4,99 in women) (Table 1, Fig. 2). Incidence of MM increases gradually from age 55 in both sexes (Fig. 3), and cumulative rate for men is estimated at 0.77. LMNH are the second most frequent HM. They increase with age, after 35 years, for both sexes, particularly for men, with a higher standardized incidence than women (5.93 per 100,000 as compared to 5.05 for women). (Table 1, Fig. 3). LAM have a higher incidence in men than in women (2.19 per 100,000 versus 1.38) and increases from the age of 70 years for men (Table 1, Fig. 3). The less frequent HM are LH, with the lowest 10-year incidence. MM, LMNH and LAM are diagnosed among older people more frequently (median age of both sexes is over 68) in contrast to LH, which have median age at diagnosis of fewer than 40 years (Table 1).

**Fig. 4:**
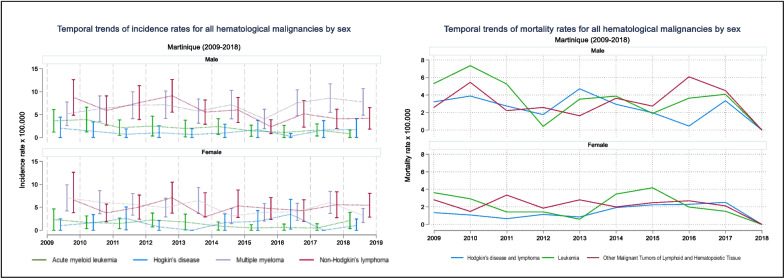
Temporal Trends of standardized incidence and mortality rates for all Hematological Maligancies by sex, in Martinique (2009-2018)

Temporal distribution of trends in standardized incidence of all HM decreased overall in both sexes, except for MM in men (Fig. 4). MM showed a substantial increase from 2015 in men (4.04 in 2015 vs 8.58 in 2017) (Fig. 4).

In both sexes, the evolution of standardized incidence rates of LH and LAM are constant and do not exceed 4 per 100,000 (Fig. 3). LMNH have a similar evolution in both sexes but less in women. In men, rates are above 5 per 100,000 until 2014, with a peak in 2012 of 9.10 per 100.000, and a decrease in 2015 to remain below 5 (Fig. 3).

MM have rates between 6.3 and 7.2 per 100,000 between 2010–2014 for males, increasing in 2015 to a peak in 2017 of 8.6. Similar evolution is observed in women but with lower rates, close to 5 per 100,000 (Fig. 4).

### Mortality of Hematological Malignancies in Martinique

CépiDC data showed highest mortality rates for men and significant variability in mortality rates for both sexes, with SMR above 8.5 per 100,000 for leukemia and other malignant tumors of lymphoid and hematopoietic tissue. Hodgkin's disease and lymphomas have the lowest SMR, with higher mortality in men (6.02 per 100,000 versus 3.23) (Table 2). In addition, temporal trends of mortality rates for all HM has decreased overall (Fig. 4).

Before and including 2012, males have mortality rates for Hodgkin's disease and lymphoma between 5 and 8 per 100,000. There was an initial peak in 2013 and a steady decline through 2016 (12.14 per 100,000 in 2013 versus 1.13 per 100,000 in 2016). Mortality rate increased slightly by six units in 2017. In women, Hodgkin's disease and lymphoma are distributed differently over time. Mortality rates were generally less than 5 per 100,000 before and including 2016 and peaked in 2017 at 6.87 per 100,000 (Fig. 4).

For leukemias, temporal trends are different in both sexes. In women, there was a slight decrease in the mortality rate until 2013 (7.16 per 100,000 in 2009; 2.50 per 100,000 in 2013). The rates then increase slightly between 2014 and 2017, reaching a rate close to 10 per 100,000. Among men, mortality rates fluctuate over the period for leukemia. Until 2011, the rates were above 10 per 100,000 (with a peak of 18.33 per 100,000 in 2010), then a decrease in rates until reaching 1.5 per 100,000 in 2012, continuing by an increase until 2017 with rates above 10 (Fig. 4).

In contrast to men, the mortality rates of other malignant tumors of lymphoid and hematopoietic tissues have a stable evolution between 5 and 8 per 100,000 in women. In men, there was a peak at 12.98 per 100,000 in 2010, then maintained between 6 and 7 per 100,000 between 2012 and 2015, with a sudden increase to a mortality rate equal to 13.9 per 100,00 in 2016 (Fig. 4).

### Standardized incidence specific rates by age and gender

Gender-specific rates showed a multimodal distribution curve that increased with age for all HM (Fig. 3). Predominance was in male for all cases of HM in all age groups. Extreme age range was 1 to 98 years.

We observed a comparable evolution of the overall trend in incidence for LMNH according to age in men and women. However, aged men are more affected than women. Age-specific incidence rate curve showed a steady and apparent increase in rates after age 50 in both sexes (Fig. 3), reaching a first frequency peak for 65–69 years in females and a maximum value between the ages of 70–84 years to reach a rate of 46.25 in males and 38.80 in females (Fig. 3).

LH were observed at all ages, particularly in younger subjects (< 45 years) (Fig. 3). Age-specific incidence curve for men showed a first frequency peak for 20–29 years (SIR: 3.6), a second frequency peak was observed for 35–39 years (SIR: 3.3), a maximum frequency peak for 80–85 years with a SIR reaching 3.1 (Fig. 3). In women, age-specific incidence rate curve showed a maximum frequency peak for 25–34 years (SIR: 4.99; 3.78), a second frequency peak was observed for 40–54 years (SIR: 1.8), and a final frequency peak for 75–79 years (SIR: 1.36) (Fig. 3).

We observed similar trend in the overall incidence of MM by age in men and women. The incidence rate curve by age and sex showed that a few cases of multiple myeloma were observed before age 40. The increase in frequency is steady and continuous, with a significant increase after 55 years of age with a maximum frequency of 80–85 years (102.55 for men; 76.22 for women) (Fig. 3).

Frequency of LAM also varied with age. Only one case was observed before the age of 10 years in women (Fig. 3). Incidence rate curve by age and sex showed that the increase in the frequency of LAM is regular and continuous, with a more significant evolution for men from the age of 70 (34.18 per 100.000 for men; 10.64 per 100.000 for women) (Fig. 3).

## Discussion

We described the characteristics of an epidemiological analysis of HM based on a population-based cancer registry in the French West Indies from 2009 to 2018. It is a main strength to carry out this work using data from GCRM, which is well established in the health care systems and essential in Caribbean public health.

In Martinique, incidence of all cancers combined was currently lower than mainland France but was following a negative trend, probably due to the aging population and the increased prevalence of lifestyle risk factors (sedentary lifestyle, overweight and obesity, smoking). According to the GCRM, more than 1,583 new cases of invasive cancer are recorded each year, with a male/female ratio of 1.5. Incidence of cancer observed from 2009 to 2018 differs from that of France, as higher incidences have been observed for MM in particular. However, median ages are similar [9].

Over the study period in Martinique, temporal standardized incidence distribution of all HM had decreased and persistent after 2013 for all HM groups except for MM, which showed a notable increase in incidence since then. In Martinique, the incidence was still twice as high as in France [9]. Our study showed that LMNH was diagnosed twice less often than in mainland France. LMNH was known to be 2.5 times more common in developed countries, particularly in France, which was among the European countries with the highest incidence [11]. The year 2000 marked the beginning of the decline in the incidence of LMNH in mainland France. In Martinique, this decline was observed more recently in 2013 [9, 13].

Concerning worldwide epidemiology, HM was the fourth most common location and the sixth leading cause of death for all cancers combined. Our results would reflect significant points of convergence with what was observed in the Pan-American area in the United States and in the rest of the world. In particular, male dominance of incidence and mortality was observed in all age groups. Incidence rates for LMNH, LH, and MM can range from 20 to 30 worldwide [14–16]. LH incidence was higher in developed countries. Incidence by age varies between the industrialized countries of the Western world and the so-called developing countries [10]. MM incidence rates were higher for both sexes in black African-descent populations in France and in the United States [5].

More recently, a significant and original study used 2012–2017 vital statistics data and cancer mortality data from four states: California, Florida, Minnesota, and New York. These states provided a balance of West Africans, more common in New York, with East Africans, more common in Minnesota, and African-Caribbean, found mainly in Florida and New York. African-Americans born in the United States showed highest burden of cancer mortality for MM and LAM [17]. In this study, there was a descending gradient in mortality for HM with a sex ratio in favor of males: African-American, African-Caribbean, and African. Analysis showed several similarities with this study of subgroups of African-Americans in the United States, namely a similar distribution in the frequency of diagnosed HM (MM, LAM, LMNH, and very few LH), associated with standardized incidence and mortality rates of MM that were among the highest in the world. It should be noted, however, that MM mortality rates observed in Martinique in men and women were systematically lower in comparison with the three subgroups of black ethnic groups in the USA [9, 10, 16–18].

Available data from the Pan American zone is vital because it allows the GCRM to compare neighboring populations with shared cultures, customs, and origins. In Latin America, only 8% of population were covered by cancer registries [19]. HOLA, a multi-center retrospective observational study (Hemato-Oncology Latin America), generated unprecedented data on patient's characteristics and treatment patterns with HM [20]. Distribution frequencies and pathology characteristics were comparable to those observed in Europe or Asia and, therefore, different from those observed in Martinique and black populations since 57.7% of patients had LMNH, 29.5% had MM, and 12.7% had LAM. This work revealed that the median age of patients was younger than those observed in Martinique and France for MM (67.4 versus 72 years) and LMNH (58 versus 62 years) and comparable for LH and LAM. There was a slight predominance of males (54.2%) over females (45.8%), except for LMNH, which had a sex ratio in favor of females. The most frequent age categories for all subtypes of HM were also comparable with those observed in Martinique. As in the rest of the world, regional disparities were observed in this Pan-American region [20]. Incidence and mortality rates were intermediate between Caucasian and Black populations in the USA [9, 20].

According to the literature in Martinique and mainland France, LH was a common pathology in young people; the median age is 39 in men and 33 in women. As observed in most African-descent populations worldwide, LH incidence rates in Martinique were lower (men/women: 1.2/1.4) than in France and Europe [5, 9, 10]. Socioeconomic status has been shown to influence the epidemiology of LH. These observations led to the formulation of a late infection model, in which the absence of exposure to infectious agents in childhood increases the risk of LH in young adults [21]. Concerning familial risk, epidemiological studies have shown that the risk is fourfold in first-degree relatives of LH patients [22]. Other factors are also thought to increase the risk of LH, including eczema and autoimmune diseases [23].

As observed in mainland France and more widely, LAM in Martinique was rare and mainly affected elderly subjects. Sex ratio reflecting similar incidence. Incidence rates were low and relatively stable over the study period as those observed in mainland France until 2010 [9, 24] or United States [25].

The origin of LMNH was multifactorial, involving genetic, viral, and environmental factors. The known risk factors for LMNH were primary immune deficiencies, organ transplants, and infectious agents such as Helicobacter Pylori, hepatitis C virus or T-cell lymphomas due to the human lymphotropic virus T-HTLV-1, autoimmune diseases such as Sjögren's syndrome and systemic lupus erythematous, family and personal history of HM [26, 27].

Meta-analysis confirmed a link between occupational exposure to pesticides and LMNH subtypes [28–30]. The French West Indies were characterized by high pesticide exposure, particularly to chlordecone (CLD), an insecticide used in banana plantations. MM, 1st HM in Guadeloupe and Martinique, was over-incidence compared to France. Information on pollutant contamination at an acceptable geographical level was available in the French West Indies. As part of the Chlordecone Plan implemented throughout the French West Indies, a study of the spatial distribution of cancers potentially linked to soil pollution by organochlorine pesticides was carried out in 2008 by the GCRM, with the support of the Regional Health Agency of Martinique. The study showed an excess incidence of MM in men living in communities with the highest levels of soil contamination, suggesting occupational exposure to organochlorine pesticides. Work on the banana worker cohort found a non-significant excess of deaths from non-Hodgkin's lymphoma and significant for multiple myeloma [24, 31].

Overall incidence rate of myeloma has increased due to an aging population. MM were among the HM with higher incidence and mortality in French West Indies territories than in mainland France. Estimated incidence of MM in 2018 in France was slightly above the European average [32] and slightly lower than in the United States [25]. According to the literature, it was well known that this over incidence could be related to ethnicity.

Future epidemiological studies should allow the declination of the characteristics of the most frequent subtypes of HM in the Caribbean region and their degrees of heterogeneity. Nevertheless, all these results suggest that modern adapted therapies can increase the survival time of patients, especially for MM. For instance, hematopoietic stem cell autotransplantation by cryopreservation of grafts obtained by cytapheresis provides comprehensive access to care for patients with severe forms of myeloma and non-Hodgkin's lymphoma. This therapeutic procedure is complementary to high-dose chemotherapy, depending on the clinical indications, with preventive or curative aims [33–36].

## Conclusion

The results of this work would show a change in the evolution of the epidemiology of HM in Martinique with a clear downward trend in temporal trends, which has been confirmed in recent years. We also noted similarities with populations of African origin in the United States or the Pan-American zone, such as higher standardized incidence rates than in the rest of the world. Indeed, we have found a similar distribution of subtypes for HM associated with similar over-incidences. Differences in treatment and access to specific innovative techniques or therapeutic lines could explain these changes.

Ethno-geographical and socio-economic characteristics of this predominantly Afro-Caribbean population might explain some of its disparities. In addition, the role of infectious agents and environmental factors specific to the Caribbean region warrants specific research with neighboring territories that could help identify other risk factors. Finally, genome-wide association studies should also be implemented in the Caribbean to identify genetic variants that would influence the risk of genetic predisposition.

Conducting original studies in the Caribbean, characterized by atypical epidemiology and ecosystem, would provide a wide range of information on environmental exposure, co-infections with emerging viruses such as COVID-19, and the genetic variants of risk involved. Our results will contribute to expanding knowledge on the epidemiology of heterogeneous diseases such as HM disease in the Caribbean zone with a primary objective; to develop a regional strategy integrating aspects related to the patient's quality of life.

## Data Availability

The datasets generated and/or analysed during the current study are not publicly available due to French legislation (Cancer data is code-anonymized and cannot be shared without authorization) but are available from the corresponding author on reasonable request.

## Abbreviations

CLD: Chlordecone
CépiDc: French Epidemiology Center on Medical Causes of Death
ENCR: European Network of Cancer Registries
EU: European Union
GCRM: General Cancer Registry of Martinique
GBD: Global Burden of Disease
GDPR: General Data Protection Regulation
HM: Hemopaties Malignancies
HOLA: Hemato-Oncology Latin America
HTLV-1: Human T-cell lymphotropic virus type 1
IARC: International Agency for Research on Cancer
LMNH: Non-Hodgkin’s Lymphomas
LAM: Acute Myeloid Leukemia
LH: Hodgkin’s disease
MM: Multiple Myeloma
SIR: Standardized Incidence rates
SMR: Standardized Mortality rates
USA: United States of America
US: United States
WHO: World Health of Organization

## References

[1] Chan JK (2001). The new World Health Organization classification of lymphomas: the past, the present and the future. Hematol Oncol.

[2] Jaffe ES, Harris NL, Stein H, Vardiman J (2001). Pathology and Genetics of Tumours of Haematopoietic and Lymphoid Tissues.

[3] Lee H, Park HJ, Park EH, Ju HY, Oh CM, Kong HJ (2018). Nationwide statistical analysis of lymphoid malignancies in Korea. Cancer Res Treat.

[4] Liu W, Liu J, Song Y, Zeng X, Wang X, Mi L (2019). Burden of lymphoma in China, 2006–2016: an analysis of the global burden of disease study 2016. J Hematol Oncol.

[5] Waxman AJ, Mink PJ, Devesa SS, Anderson WF, Weiss BM, Kristinsson SY (2010). Racial disparities in incidence and outcome in multiple myeloma: a population-based study. Blood.

[6] Clarke CA, Glaser SL, Gomez SL, Wang SS, Keegan TH, Yang J (2011). Lymphoid malignancies in U.S. Asians: incidence rate differences by birthplace and acculturation. Cancer Epidemiol Biomarkers Prev.

[7] Rosenberg PS, Barker KA, Anderson WF (2015). Future distribution of multiple myeloma in the United States by sex, age, and race/ethnicity. Blood.

[8] Tan D, Horning SJ, Hoppe RT, Levy R, Rosenberg SA, Sigal BM (2013). Improvements in observed and relative survival in follicular grade 1–2 lymphoma during 4 decades: the Stanford University experience. Blood.

[9] Joachim-Contaret C, Veronique-Baudin J, Macni J, Ulric-Gervaise S, Cariou M, Billot-Grasset A (2019). Estimations régionales et départementales d’incidence et de mortalité par cancers en France, 2007-2016.

[10] Bray F, Ferlay J, Soerjomataram I, Siegel RL, Torre LA, Jemal A (2018). Global cancer statistics 2018: GLOBOCAN estimates of incidence and mortality worldwide for 36 cancers in 185 countries. CA Cancer J Clin.

[11] Ferlay J, Soerjomataram I, Dikshit R, Eser S, Mathers C, Rebelo M (2015). Cancer incidence and mortality worldwide: sources, methods and major patterns in GLOBOCAN 2012. Int J Cancer.

[12] Boyle P, Parkin DM. Chapter 11. Statistical methods for registries. Cancer Registration: Principles and Methods. 95. Lyon: IARC Scientific Publication; 1991. 1894318

[13] Belot A, Grosclaude P, Bossard N, Jougla E, Benhamou E, Delafosse P (2008). Cancer incidence and mortality in France over the period 1980–2005. Rev Epidemiol Sante Publique.

[14] Orem J, Mbidde EK, Lambert B, de Sanjose S, Weiderpass E (2007). Burkitt's lymphoma in Africa, a review of the epidemiology and etiology. Afr Health Sci.

[15] Iwanaga M, Watanabe T, Yamaguchi K (2012). Adult T-cell leukemia: a review of epidemiological evidence. Front Microbiol.

[16] Besson C, Gonin C, Brebion A, Delaunay C, Panelatti G, Plumelle Y (2001). Incidence of hematological malignancies in Martinique, French West Indies, overrepresentation of multiple myeloma and adult T cell leukemia/lymphoma. Leukemia..

[17] Pinheiro PS, Medina H, Callahan KE, Kwon D, Ragin C, Sherman R (2020). Cancer mortality among US blacks: Variability between African Americans, Afro-Caribbeans, and Africans. Cancer Epidemiol.

[18] Carreon JD, Morton LM, Devesa SS, Clarke CA, Gomez SL, Glaser SL (2008). Incidence of lymphoid neoplasms by subtype among six Asian ethnic groups in the United States, 1996–2004. Cancer Causes Control.

[19] Forman DBF, Brewster DH, Gombe Mbalawa C, Kohler B, Piñeros M, Steliarova-Foucher E, Swaminathan R, Ferlay J (2014). Cancer Incidence in Five Continents Volume X: IARC Scientific Publication No. 164.

[20] de TietscheMoraes Hungria V, Chiattone C, Pavlovsky M, Abenoza LM, Agreda GP, Armenta J (2019). Epidemiology of hematologic malignancies in real-world settings: findings from the hemato-oncology Latin America observational registry study. J Glob Oncol.

[21] Cozen W, Hamilton AS, Zhao P, Salam MT, Deapen DM, Nathwani BN (2009). A protective role for early oral exposures in the etiology of young adult Hodgkin lymphoma. Blood.

[22] Goldin LR, Bjorkholm M, Kristinsson SY, Turesson I, Landgren O (2009). Highly increased familial risks for specific lymphoma subtypes. Br J Haematol.

[23] Kristinsson SY, Landgren O, Sjoberg J, Turesson I, Bjorkholm M, Goldin LR (2009). Autoimmunity and risk for Hodgkin's lymphoma by subtype. Haematologica.

[24] Guyader-Peyrou SL, Defossez G, Dantony E, Mounier M, Cornet E, Uhry Z, et al. Estimations nationales de l’incidence et de la mortalité par cancer en France métropolitaine entre 1990 et 2018. Étude à partir des registres des cancers du réseau Francim. Volume 2 - Hémopathies malignes. 2019.

[25] Noone AM HN, Krapcho M, Miller D, Brest A, Yu M, Ruhl J, Tatalovich Z, Mariotto A, Lewis DR, Chen HS, Feuer EJ, Cronin KA. SEER Cancer Statistics Review, 1975–2015, National Cancer Institute. Bethesda, MD, based on November 2017 SEER data submission, posted to the SEER web site, April 2018. 2018 [Available from: https://seer.cancer.gov/csr/1975_2015/.

[26] Alexander DD, Mink PJ, Adami HO, Chang ET, Cole P, Mandel JS (2007). The non-Hodgkin lymphomas: a review of the epidemiologic literature. Int J Cancer.

[27] Ekstrom-Smedby K (2006). Epidemiology and etiology of non-Hodgkin lymphoma–a review. Acta Oncol.

[28] Morton LM, Slager SL, Cerhan JR, Wang SS, Vajdic CM, Skibola CF (2014). Etiologic heterogeneity among non-hodgkin lymphoma subtypes: the InterLymph non-hodgkin lymphoma subtypes project. J Natl Cancer Inst Monogr.

[29] Morton LM, Sampson JN, Cerhan JR, Turner JJ, Vajdic CM, Wang SS (2014). Rationale and design of the international lymphoma epidemiology consortium (InterLymph) non-hodgkin lymphoma subtypes project. J Natl Cancer Inst Monogr.

[30] IARC. IARC Monographs on the identification of carcinogenic hazards to humans 2022 [updated 2022–09–07 10.34am (CEST). Agents classified by the IARC Monographs, Volumes 1–132]. Available from: http://monographs.iarc.fr/ENG/Classification/latest_classif.php.

[31] Dieye A, Quénel P, Goria S, Blateau A, Colonna M, Azaloux H (2019). Étude de la répartition spatiale des cancers possiblement liés à la pollution des sols par les pesticides organochlorés, en Martinique.

[32] Ferlay J, Colombet M, Soerjomataram I, Dyba T, Randi G, Bettio M (2018). Cancer incidence and mortality patterns in Europe: Estimates for 40 countries and 25 major cancers in 2018. Eur J Cancer.

[33] Jansen J, Nolan PL, Reeves MI, Akard LP, Thompson JM, Dugan MJ (2009). Transportation of peripheral blood progenitor cell products: effects of time, temperature and cell concentration. Cytotherapy.

[34] Passweg JR, Baldomero H, Bregni M, Cesaro S, Dreger P, Duarte RF (2013). Hematopoietic SCT in Europe: data and trends in 2011. Bone Marrow Transplant.

[35] Hubel K, de la Rubia J, Azar N, Corradini P (2015). Current status of haematopoietic autologous stem cell transplantation in lymphoid malignancies: a European perspective. Eur J Haematol.

[36] ANSM: Agence nationale des produits de santé. "Rapport d’activité biovigilance 2011". 2012;31–32.

